# Cytokine-mediated inhibition of *Staphylococcus aureus* adherence and invasion into nonphagocytic cells

**DOI:** 10.1007/s00430-025-00840-4

**Published:** 2025-06-20

**Authors:** Arif Luqman, Knut Ohlsen

**Affiliations:** 1 Biology Department, Institut Teknologi Sepuluh Nopember, Surabaya, Indonesia; 2 Institute of Molecular Infection Biology, University of Würzburg, 97080 Würzburg, Germany

**Keywords:** Bacterial adherence, Bacterial invasion, Cytokines, Receptors for adhesin and invasin, Staphylococcus aureus

## Abstract

*Staphylococcus aureus* is a major pathogen responsible for a wide range of infections, from minor skin diseases to life-threatening conditions, such as sepsis and pneumonia. Its ability to invade nonphagocytic cells, evading the immune system and persisting intracellularly complicates the treatment and contributes to recurrent infections. In this study, we investigated the role of cytokines in inhibiting *S. aureus* adherence and invasion into nonphagocytic human cells. Monomac-6 cells were stimulated with heat-killed *S. aureus* (HKSA) to produce a cytokine cocktail, which was used to treat various human cell lines, including HEK293, A549, HaCaT, and HT29. Our results demonstrate that cytokines significantly reduced *S. aureus* adherence and invasion into HEK293, HaCaT, and HT29 cells by altering the expression of key host cell receptors for *S. aureus* adhesins and invasion, such as integrins and heat shock proteins. These effects of cytokines were mediated via JAK-STAT pathway as tofacitinib supplementation, a JAK inhibitor, reversed the effects of cytokine cocktail. However, these effects were not observed in A549 cells, most likely due to their ability to actively internalize pathogens. These findings suggest that cytokines provide a crucial line of defense against the ability of *S. aureus* to invade nonphagocytic cells by modulating the expression of host cells receptors.

**Supplementary Information:**

The online version contains supplementary material available at 10.1007/s00430-025-00840-4.

## Introduction

*Staphylococcus aureus* is an opportunistic and notorious pathogen responsible for a wide range of diseases, from superficial infections on the skin to life-threatening conditions such as sepsis [1]endocarditis [2, 3]osteomyelitis [4, 5]and pneumonia [6–8]. The increasing prevalence of antibiotic-resistant strains of *S. aureus*, including methicillin-resistant *S. aureus* (MRSA) [9, 10]worsens the burden of *S. aureus* infections, complicating the effective treatment against the pathogen. While our immune system provides a robust defense mechanism against *S. aureus* infections, including the inflammatory response upon the detection of *S. aureus* or its virulence factors by releasing cytokines to recruit and activate immune cells, *S. aureus* has evolved sophisticated strategies to evade the immune response to persist within the host [11, 12].

One of the primary immune evasion strategies evolved by *S. aureus* is the invasion of nonphagocytic cells [13, 14]. Invasion of *S. aureus* into nonphagocytic cells allows the bacteria to escape and hide from the immune surveillance and phagocytosis, which would make the infections difficult to eradicate and prone to recurrence [14–16]. Adherence on the host cells is the crucial initial step for *S. aureus* to invade nonphagocytic cells, mediated by bacterial surface proteins known as adhesins and invasins [17–19]. This step primarily involves fibronectin-binding proteins (FnBPs) on the surface of *S. aureus* interacting with α5β1 integrin of the host cells using fibronectin as a bridge in between 20 and [23]. Besides, there are many other mechanisms elucidated as secondary mechanisms, which involve clumping factor A (ClfA), serine aspartate repeat-containing protein D (SdrD), serine-rich adhesin for platelets, and the major autolysin (Atl) [18, 24]. Each of these microbial surface components recognizing adhesive matrix molecules (MSCRAMMs) and Atl have their specific receptors from the host cells, namely SdrD binds to Desmoglein 1 [25,26]; ClfA binds to αVβ3 integrin using fibrinogen as a bridge or complex bridge involving von Willebrand factor [27, 28]; SraP binds to gp340 [29]; Atl interacts with Hsc70 [30]; lipoprotein-like lipoproteins (Lpls) bind to Hsp90 [31], in which the interactions between them mediate the adherence and invasion of *S. aureus* into host cells by promoting the cytoskeletal reorganization that allows the bacterial cells engulfment by the nonphagocytic cells, enabling *S. aureus* to persist intracellularly. Not only MSCRAMMs, secreted trace amines, which were reported to be produced in some staphylococci [32, 33]could facilitate bacterial invasion into α2-adrenergic receptor-expressing nonphagocytic cells [34].

In response to *S. aureus* infection, the host innate immune system releases a wide array of cytokines, which are crucial for initiating and regulating inflammatory responses in *S. aureus* infections, including interleukin-1β (IL-1β), interleukin-6 (IL-6), interleukin-8 (IL-8), gamma interferon (IFNγ), and tumor necrosis factor α (TNFa) [35–40]. They modulate the body’s defense mechanisms by recruiting more immune cells to the infection site to promote pathogen eradication [41]. The released cytokines also affect surrounding tissues, by promoting increased vascular permeability and cellular adhesion [42] and the production of antimicrobial peptides [43]to help contain the infection and mitigate the damage caused by *S. aureus* infection.

However, *S. aureus* has evolved various mechanisms to invade host cells and hide from extracellular immune surveillance; protection from antibodies, complement, and phagocytic cells. The internalized *S. aureus* are then capable of exploitating the host cell machinery for survival and even replication, which might lead to latent or chronic infection [44–48]. In addition to being protected by the host immune defense, bacteria internalized into the host cells are also protected from some classes of antibiotics [49, 50]. The internalized *S. aureus* could also lead to recurrent infection, tissue damage via the induction of apoptosis and the exacerbation of the disease [46, 51–54].

With the sophisticated strategy used by *S. aureus* to survive inside the host, the host itself develops mechanisms to hinder *S. aureus* from invading the host cells. This study aimed to investigate the influence of cytokines on *S. aureus* adherence and invasion into nonphagocytic cells. Using cytokine cocktails released by heat-killed *S. aureus* (HKSA)-stimulated Monomac-6 cells, we investigated the impact of cytokines on the adherence and invasion of *S. aureus* in various nonphagocytic human cell lines, including keratinocytes (HaCaT), alveolar epithelial cells (A549), kidney cells (HEK293), and colon epithelial cells (HT29). We also assessed the expression level of host cell receptors for *S. aureus* adhesins and invasins to elucidate the modulation of cytokines on *S. aureus* adherence and invasion into nonphagocytic cells. These findings provide novel insights into the immune-mediated defense mechanisms against *S. aureus* invasion.

## Materials and methods

### Cell and bacterial culture conditions

In this study, we used 4 cell lines: HaCaT, A549, HEK293, HT29, and Monomac-6, which were kindly provided by Prof. Dr. Friedrich Götz and were originally obtained from DSMZ, Germany. These cell lines, except for Monomac-6, were cultured in DMEM with 10% fetal bovine serum (FBS) and an antibiotic mixture of penicillin‒streptomycin (Gibco™, Thermo Fisher Scientific, Germany) and incubated in a humidified incubator at 37 °C in 5% CO_2_. The difference for Monomac-6 is that we used RPMI (Gibco™, Thermo Fisher Scientific, Germany) supplemented with OPI medium supplementation (Sigma Aldrich, Germany). We used *S. aureus* USA300 LAC and *S. aureus* Cowan as model strains in this study. These *S. aureus* strains were grown in basic media (BM) (1% soy peptone, 0.5% yeast extract, 0.5% NaCl, 0.1% glucose, and 0.1% K_2_HPO_4_, pH 7.2) at 37 °C under continuous shaking at 200 rpm.

### Heat-killed *S. aureus* preparation

We prepared heat-killed *S. aureus* USA300 LAC and *S. aureus* Cowan for immune cell stimulation. The bacterial strain was inoculated in BM and incubated overnight (16 h) at 37 °C with shaking at 200 rpm. The overnight culture was then pelleted by centrifugation, washed twice, and resuspended in sterile phosphate-buffered saline (PBS). The resuspended bacterial cells were then adjusted to 3 × 10^8^ colony-forming units (CFUs)/ml and incubated at 90 °C for 20 min. The heat-killed *S. aureus* (HKSA) USA300 LAC and Cowan were then stored at -20 °C.

### Monomac-6 stimulation

The cultured Monomac-6 cells were reseeded in fresh media at a density of 10^6^ cells/ml. HKSA was then supplemented into the culture at a multiplicity of infection (MOI) of 30. Sterile PBS was used as a supplement for the control or unstimulated groups. We also used dexamethasone (Biomol GmbH, Germany) at a concentration of 50 µg/ml to suppress cytokine production. The cells were then incubated in a humidified incubator at 37 °C in 5% CO_2_ for 16 h. The supernatant was then collected by centrifugation, filtered through a 0.02 μm filter, and stored at -20 °C.

### Cytokine quantification

The cytokine concentration of the collected Monomac-6 supernatant was measured using ELISA kits. We quantified the concentrations of TNFα, IL-4, IL-6, IL-8 (Immunotools GmbH, Germany), IFNγ, IL-1β, IL-10, and IL-13 (Thermo Fisher Scientific, Germany) according to the manufacturers’ instructions for the ELISA kits.

### Adherence and invasion assays

We performed adherence and invasion assays on 4 different cell lines: HEK293, A549, HT29, and HaCaT. The invasion assays were conducted using gentamicin survival assay. The cells were seeded in a 24-well plate in fresh media with a final cell number of 10^6^ cells/well on the day of the adherence or invasion assay. One day prior to the adherence and invasion assays, for the experiments with Monomac-6 supernatant, the media of the seeded cells in the 24-well plate were exchanged with the collected Monomac-6 supernatant, whereas for the experiments with cytokines (TNFα, IFNγ, IL-1β, IL-4, IL-6, IL-8, IL-10, and IL-13 (Immunotools GmbH, Germany)), the cytokines were directly added to the wells at a final concentration of 0.5 ng/ml. The treated cells were then incubated in a humidified incubator at 37 °C in 5% CO_2_ for 16 h. The next day, the cells were washed with sterile PBS twice, and fresh DMEM without FBS and antibiotic supplement were added. The bacterial cells of *S. aureus* USA300 LAC or *S. aureus* Cowan were resuspended in sterile PBS from the overnight preculture were then added to the well at an MOI of 30 and further incubated for 1.5 h. The cells were then washed again twice with sterile PBS. For the adherence assays, 0.1% Triton X-100 was added to lyse the cells, which were then plated on agar plates after several dilutions. The inoculated agar plates were then incubated further at 37 °C overnight. The colonies grown on agar plates represent the adhered and invaded bacteria into the cells. Therefore to get the adhered bacterial number in CFU, we substracted the bacterial CFU from the adherence assay with the average value from invasion assays. For the invasion assays, after the cells were washed with sterile PBS, fresh DMEM containing 100 µg/ml gentamicin was added to the wells and further incubated at 37 °C in 5% CO_2_ for 1 h. The cells were then washed again twice with sterile PBS. For the invasion assays, 0.1% Triton X-100 was added to lyse the cells, which were then plated on agar plates after several dilutions. The inoculated agar plates were then incubated further at 37 °C overnight. The colonies grown on agar plates represent the bacteria internalized by the cells.

### Cell viability assay

HEK293, A549, HT29, and HaCaT cells were seeded in a 96-well plate in fresh media to make a final density of 10^5^ cells/well on the day of the experiment. On the day of the experiment, the cells were treated with cytokines at a final concentration of 0.5 ng/ml or exchanged with the supernatant of Monomac-6 (stimulated and unstimulated) and incubated in a humidified incubator at 37 °C in 5% CO_2_ for 16 h. The next day, iodonitrotetrazolium (INT) was added to each well at a final concentration of 4.5 µg/ml, and further incubated for 4 h at 37 °C in 5% CO_2_. Dimethylsulfoxide (DMSO) (100 µl) was then added to each well, and the mixture was incubated at room temperature for 16 h. We then measured the absorbance at 490 nm. We also performed cell viability assays for cells treated with living *S. aureus* USA300 LAC and Cowan at MOI of 30. The incubation was bacterial cells were conducted a humidified incubator at 37 °C in 5% CO_2_ for 1.5 h and gentamicin (100 µg/ml) was added and incubated further for 1 h. INT was added to each well at a final concentration of 4.5 µg/ml, and further incubated for 4 h at 37 °C in 5% CO_2_. Dimethylsulfoxide (DMSO) (100 µl) was used to solubilize formed formazan salt in the well by incubation at room temperature for 16 h. The cell viability was calculated by comparing the absorbance at 490 nm of the treated group with the untreated (control) group.

### Relative expression of receptors for *S. aureus* adhesins and invasins

HEK293, A549, HT29, and HaCaT cells were seeded in 24-well plates in fresh media at a final density of 10^6^ cells/well on the day of RNA extraction. One day prior to RNA extraction, the media of the seeded cells in the 24-well plate were exchanged with the collected Monomac-6 supernatant, and incubated further in a humidified incubator at 37 °C in 5% CO_2_ for 16 h. The next day, we extracted RNA from the cells using *Quick-*RNA Microprep Kit (Zymoresearch, Germany) according to the manufacturer’s instructions. The extracted RNA was then used for cDNA synthesis using SuperScript™ IV Reverse Transcriptase (Thermo Fisher Scientific, Germany). The synthesized cDNA was subjected to qPCR using a Biozym Blue S’Green qPCR Kit (Biozym Scientific GmbH, Germany). We analyzed the cycle threshold values of several genes: ITGB1, ITGA5, ITGB3, ITGAV, HSP90, ANXA2, HSC70, HSP60, CD36, DSG1, and VWF as well as B2M as the housekeeping gene (Primers used in this study are listed in Supplementary Table 1). The relative expression of the genes of interest was calculated using 2 − ΔΔCT method [55].

### Statistical analyses

We analyzed the obtained data using unpaired t-test and one-way ANOVA or two-way ANOVA with the Bonferroni or Dunnett correction as a post hoc analysis. Statistical analyses were performed using GraphPad Prism software, with significance defined as *p* < 0.05; n represents independent biological replicates.

## Results

### *S. aureus* exposure induces cytokine production in monomac-6 cells

Monocytes are essential players in the defense and fight against microbial pathogen infections. These cells can be activated via direct stimulation by bacterial products and release cytokines as a rapid response to microbial stimulation [56, 57]. Therefore, in this study, we used Monomac-6 cells, a human monocytic cell line, as a source of cytokine cocktail in response to microbial products.

**Fig. 1:**
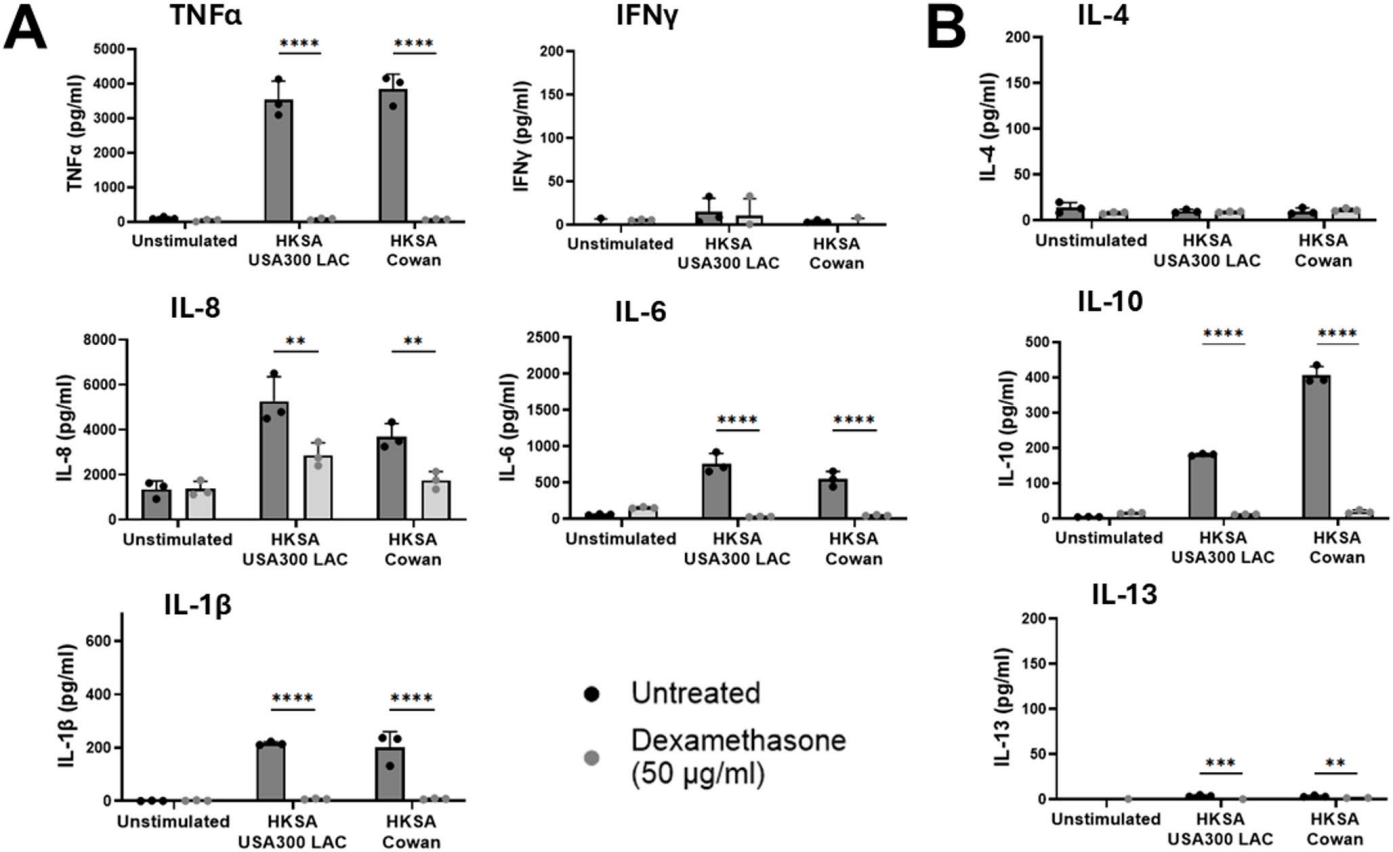
Heat-killed *S. aureus* (HKSA) USA300 LAC and Cowan induce the production of pro- and anti-inflammatory cytokines in Monomac-6 cells. Monomac-6 cells were treated with HKSA at an MOI of 30 and PBS (unstimulated) in the presence of dexamethasone (50 µg/ml) and incubated for 16 h, and cytokine production was measured using ELISA. We measured the production of proinflammatory cytokine (**A**) and anti-inflammatory cytokines **(B).** For all the graphs, each data point represents the mean value ± SD (*n* = 3). Two-way ANOVA was used to analyze the data, followed by the Bonferroni correction, with ^*^p < 0.05, ^**^p < 0.01, ^***^p < 0.001, and ^****^p < 0.0001

We stimulated Monomac-6 cells with heat-killed *S. aureus* (HKSA) USA300 LAC and Cowan at an MOI of 30 for 16 h. We also used dexamethasone, an immunosuppressive agent [58–60]together with HKSA to compare the suppression of cytokine production with that in unstimulated Monomac-6 cells. Stimulation with HKSA USA300 LAC and Cowan in Monomac-6 showed comparable cytokine profile, except for IL-10 in which HKSA Cowan induced comparatively higher IL-10 release in Monomac-6 cells compared to HKSA USA300 LAC. HKSA induced the production of several proinflammatory cytokines, such as TNFα, IL-1β, IL-8, and IL-6. TNFα and IL-8 were released into the supernatant at relatively high concentrations (in the range of thousands of pg/ml) while IL-1β and IL-6 were released into the supernatant at a comparatively lower concentration (in the range of hundreds of pg/ml) (Fig. 1A). Interestingly, Monomac-6 was also induced by HKSA to produce IL-10, an anti-inflammatory cytokine (Fig. 1B). The supplementation of dexamethasone (50 µg/ml) significantly suppressed cytokine production to the unstimulated level, except for that of IL-8, which was still twice the concentration of the IL-8 released by unstimulated Monomac-6 cells (Fig. 1).

### Cytokine cocktail from monomac-6 cells reduces *S. aureus* adherence and invasion in host cells

The supernatant containing a cocktail of cytokines produced by Monomac-6 cells from the previous experiments was used to treat the cells prior to the adherence and invasion assays. In these adherence and invasion experiments, we used 4 types of cell lines, HaCaT (an immortalized human keratinocyte cell line), A549 (adenocarcinomic human alveolar basal epithelial cells), HEK293 (human embryonic kidney cells [61]), and HT29 (a human colon cancer cell line [62]), to show that the observed effect of the cytokine cocktail treatment on bacterial adherence and invasion is not cell type specific. We used *S. aureus* as a model for the adherence and invasion assays, as the adherence and invasion mechanisms in host cells are very well studied. Moreover, the strain we used were *S. aureus* USA300 LAC because of its clinical relevance [63–65] and *S. aureus* Cowan as a reference strain widely used for FnBP-mediated invasion due to strong expression of FnBPs [21, 66].

**Fig. 2:**
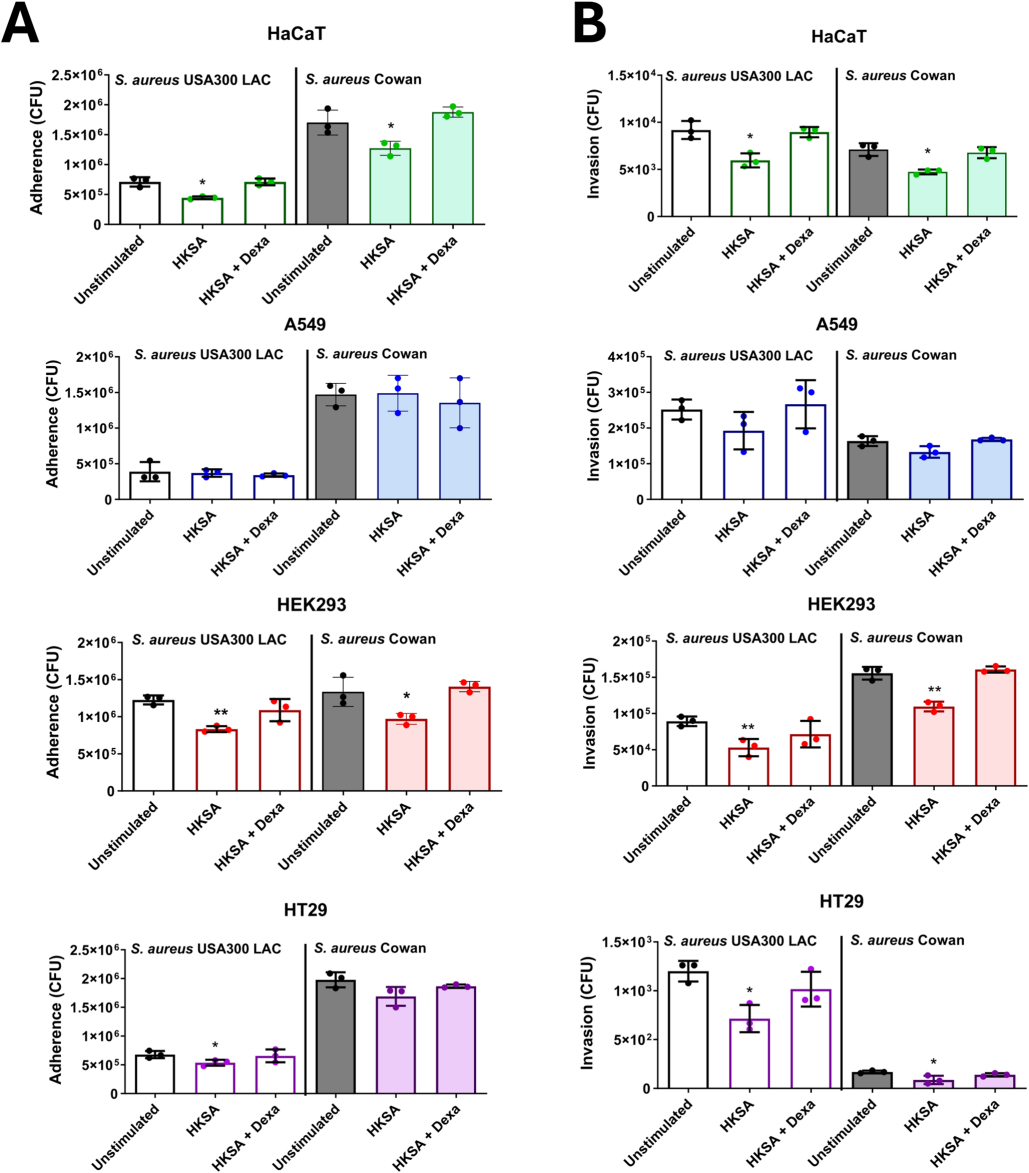
The supernatant of HKSA-stimulated Monomac-6 cells decreased bacterial adherence to and invasion into host cells. (**A**) The adherence and (**B**) invasion of *S. aureus* USA300 LAC and *S. aureus* Cowan in several cell lines (HEK293, A549, HT29 and HaCaT) were investigated after the cells were pretreated for 16 h with the supernatant of Monomac-6 cells. For all the graphs, each data point represents the mean value ± SD (*n* = 3). Unpaired t-test was used to analyze the data, with *p < 0.05 and **p < 0.01. Unstimulated: pretreated with supernatant of unstimulated Monomac-6 cells; HKSA: pretreated with supernatant of stimulated Monomac-6 cells with HKSA at MOI of 30; HKSA + Dexa: pretreated with supernatant of stimulated Monomac-6 cells with HKSA at MOI of 30 and dexamethasone (50 µg/ml)

We pretreated the cells one day prior to the adherence and invasion assays by exchanging the media with the supernatant of the unstimulated and HKSA-stimulated Monomac-6 cells with and without the addition of dexamethasone. The results of the adherence experiments revealed that, in general, the cells pretreated with the supernatant of HKSA-stimulated Monomac-6 cells showed a lower adherence of *S. aureus* compared to the cells pretreated with the supernatant of unstimulated Monomac-6 cells. The adherence level of *S. aureus* to cells pretreated with the supernatant of HKSA-stimulated Monomac-6 cells with the addition of dexamethasone was similar to that of unstimulated cells. However, interestingly, this effect was not observed particularly in the A549 cell line and in HT-29 cells when co-cultured with *S. aureus* Cowan (Fig. 2A). A similar tendency as that in the adherence experiments was also observed in the invasion experiments (Fig. 2B). The decrease in adherence and invasion was not due to cell lysis or a lower cell number after pretreatment nor bacterial exposure during adherence and invasion assays, as we conducted cell viability assays under the same conditions and under the same treatment, and we observed no significant reduction in cell viability compared with that in the untreated or control groups (Supplementary Fig. 1A & B).

We used a double control (unstimulated and HKSA-stimulated with the addition of dexamethasone) in these experiments, which suggested that the cytokine cocktail produced by Monomac-6 cells significantly decreased the adherence and invasion of *S. aureus* on and into host cells.

### Cytokines affect bacterial adherence and invasion differently by cell type

As a cytokine cocktail decreases the adherence and invasion of *S. aureus* into host cells, we further investigated which cytokines play a significant role in decreasing bacterial adherence and invasion. We pretreated the cells with a single cytokine at a concentration of 0.5 ng/ml for 16 h prior to the adherence and invasion assays. We used this concentration to mimic the physiological conditions of cytokine levels in the serum of patients with bacteremia, where the levels of IL-6 and IL-8 can reach several ng/ml and the level of TNFα can reach 1 ng/ml [67–69].

**Fig. 3:**
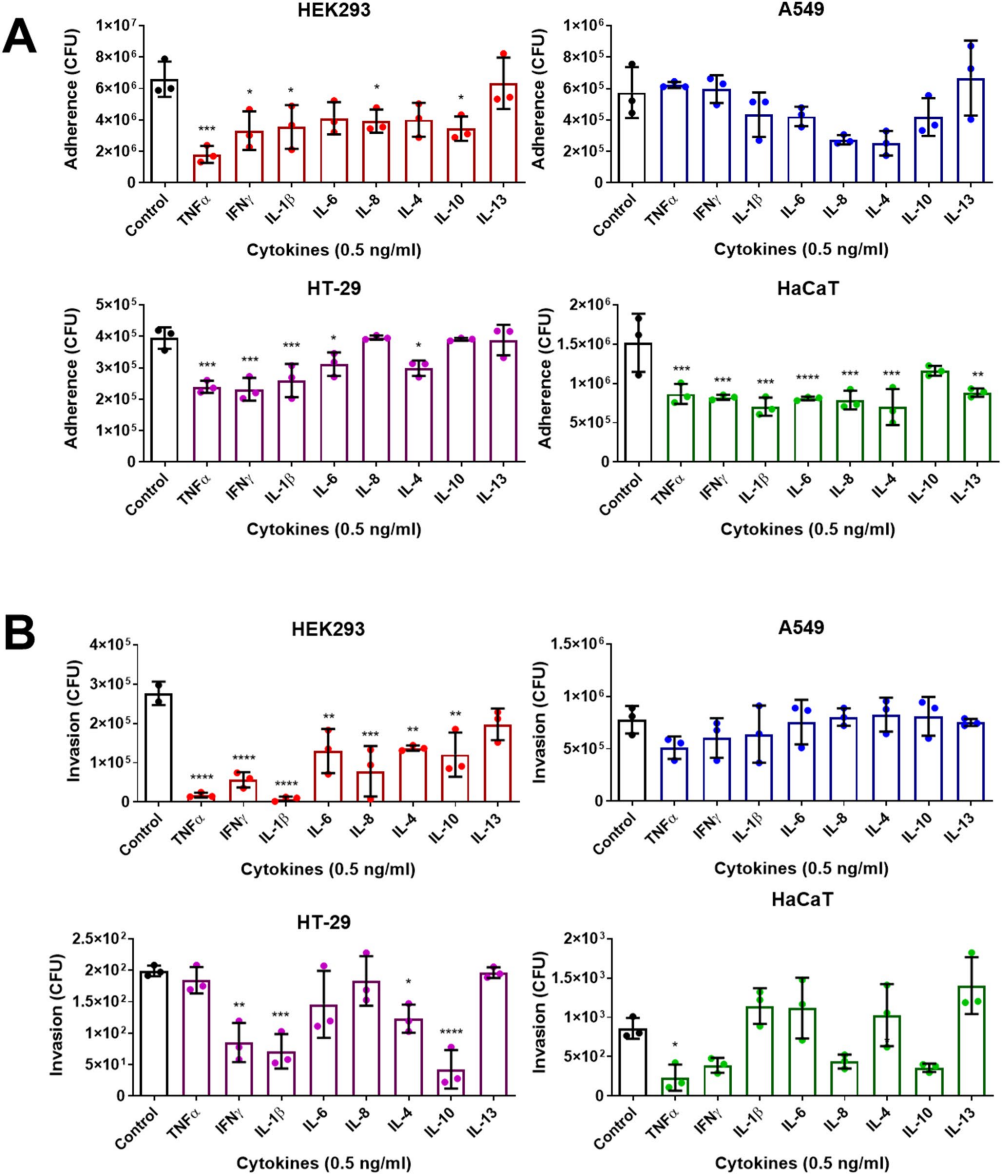
Overnight exposure of the cells to cytokines could hinder the adherence and invasion of *S. aureus * into host cells. (**A**) Adherence and (**B**) invasion assays were performed using *S. aureus* USA300 LAC on different types of cell lines (HEK293, A549, HT29, and HaCaT). The cells were pretreated for 16 h with individual cytokines (TNFα, IFNγ, IL-1β, IL-4, IL-6, IL-8, IL-10, and IL-13) at a concentration of 0.5 ng/ml prior to the assays. For all the graphs, each data point represents the mean value ± SD (*n* = 3). One-way ANOVA was used to analyze the data, followed by the Dunnett correction for multiple comparisons, with ^*^p < 0.05; ^**^p < 0.01; ^***^p < 0.001; and ^****^p < 0.0001

In these assays, we used proinflammatory (TNFα, IFNγ, IL-1β, IL-6, and IL-8) and anti-inflammatory (IL-4, IL-10, and IL-13) cytokines to treat the cells. The experiments revealed that the reduction in adherence and invasion differed among the cell types. In HEK293 cells, most of the cytokines, both pro- and anti-inflammatory, decreased the adherence and invasion of *S. aureus*, except for IL-4. In A549 cells, no cytokine significantly affected *S. aureus* adherence and invasion. In HT29 cells, TNFα and IL-6 decreased only adherence but not invasion; IL-10 decreased only invasion; and IFNγ, IL-1β, and IL-4 decreased both adherence and invasion. Moreover, in HaCaT cells, all cytokines except IL-10 decreased adherence, and TNFα was the only cytokine that significantly decreased invasion (Fig. 3). We also conducted cell viability assays on these cell lines in the presence of the cytokines, and no significant decrease in cell viability was observed (Supplementary Fig. 2). These findings suggest that the decrease in adherence and invasion in the presence of cytokines is not due to the lower viable cell number after treatment.

### Cytokine cocktail alters host cell gene expression related to bacterial adherence and invasion

**Fig. 4:**
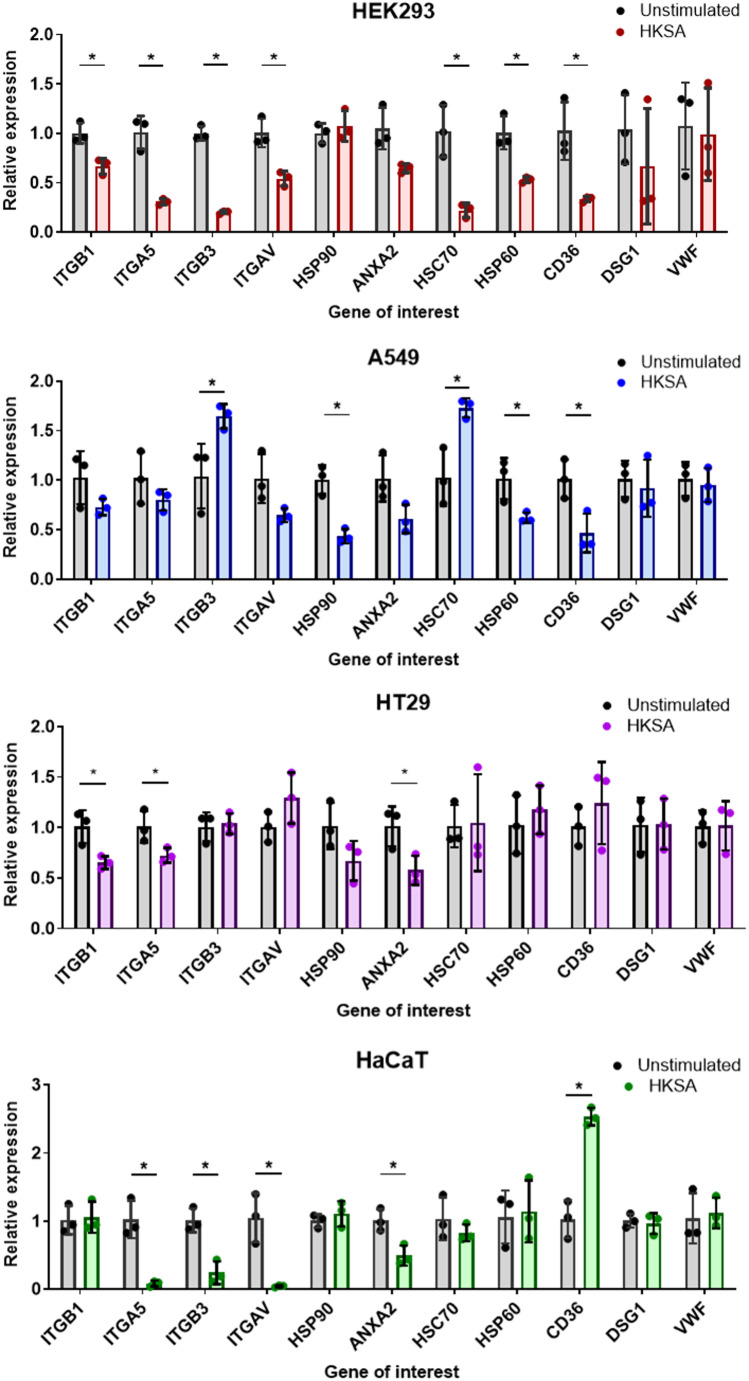
The supernatant of HKSA-stimulated Monomac-6 cells altered the gene expression of host cell receptors for *S. aureus* adhesins and invasins. The cells (HEK293, A549, HT29, and HaCaT) were treated with the supernatant of HKSA-stimulated Monomac-6 cells and the supernatant of unstimulated Monomac-6 cells and incubated for 16 h. Relative gene expression was then measured for the genes encoding receptors for *S. aureus* adhesins and invasions, namely, integrin beta 1 (ITGB1), integrin alpha 5 (ITGA5), integrin beta 3 (ITGB3), integrin alpha V (ITGAV), heat shock protein 90 (HSP90), annexin (ANXA2), von Willebrand factor (VWF), desmoglein (DSG1), heat shock protein 60 (HSP60), heat shock protein 70 (HSC70), and CD36. Beta-2-microglobulin (B2M) was used as a reference gene. For all the graphs, each data point represents the mean value ± SD (*n* = 3). An unpaired t test was used to analyze the data between unstimulated and HKSA-stimulated data with ^*^p < 0.05

The ability of *S. aureus* to adhere and to invade host cells is due to the affinity of its adhesins and invasins for their corresponding binding partners or receptors, which are present on the host cell surface [18, 31]. As the cytokine cocktail produced by Monomac-6 cells hindered bacterial adherence and invasion, we used the supernatant of unstimulated and HKSA-stimulated Monomac-6 cells to treat the cells for 16 h and measured the expression of the receptors for *S. aureus* adhesins and invasins. We measured the expression of the genes encoding integrin beta 1 (ITGB1), integrin alpha 5 (ITGA5), integrin beta 3 (ITGB3), integrin alpha V (ITGAV), heat shock protein 90 (HSP90), annexin (ANXA2), von Willebrand factor (VWF), desmoglein (DSG1), heat shock protein 60 (HSP60), heat shock protein 70 (HSC70), and CD36. We used the beta-2-microglobulin (B2M)-encoding gene as a reference. The treatment of the cells with the supernatant of HKSA-stimulated Monomac-6 cells altered the gene expression of some receptors compared with that of the cells treated with the supernatant of unstimulated Monomac-6 cells (control), and the expression alterations varied across the cell types. Compared with those in control cells, the expression levels of ITGB1, ITGA5, ITGB3, ITGAV, HSC70, HSP60, and CD36 in HEK293 cells were significantly lower. In A549 cells, the expression of HSP90, HSP60 and CD36 was lower than that in the control, and interestingly, the expression of ITGB3 and HSC70 was greater than that in the control. In HT29 cells, we observed a significant decrease in the expression of ITGB1, ITGA5, and ANXA2. Compared with those in the control cells, the expression of ITGA5, ITGB3, ITGAV, and ANXA2 in HaCaT cells was significantly lower and that of CD36 was higher (Fig. 4). These results suggest that the cytokine cocktail produced by Monomac-6 cells after stimulation with HKSA altered the expression of the receptors for *S. aureus* adhesins and invasins.

### Tofacitinib abrogates the cytokine-mediated decrease in bacterial adherence and invasion

Previous experiments showed that pretreatment of cells with the supernatant from HKSA-stimulated Monomac-6 cells led to a decrease in the adherence and invasion of *S. aureus* (Fig. 2) by altering the expression of the receptors for adhesins and invasins (Fig. 4). The supplementation of dexamethasone, which suppresses cytokine production in Monomac-6 cells (Fig. 1), reversed these effects to the control level, suggesting that the effects were due to exposure to the cytokine cocktail released by HKSA-stimulated Monomac-6 cells. As cytokines interact with and stimulate kinase activity via Janus kinases (JAKs) [70, 71]we used tofacitinib, an antagonist of several JAKs [71, 72]to investigate whether the impact observed on the treated host cells was due to the activity of cytokines.

**Fig. 5:**
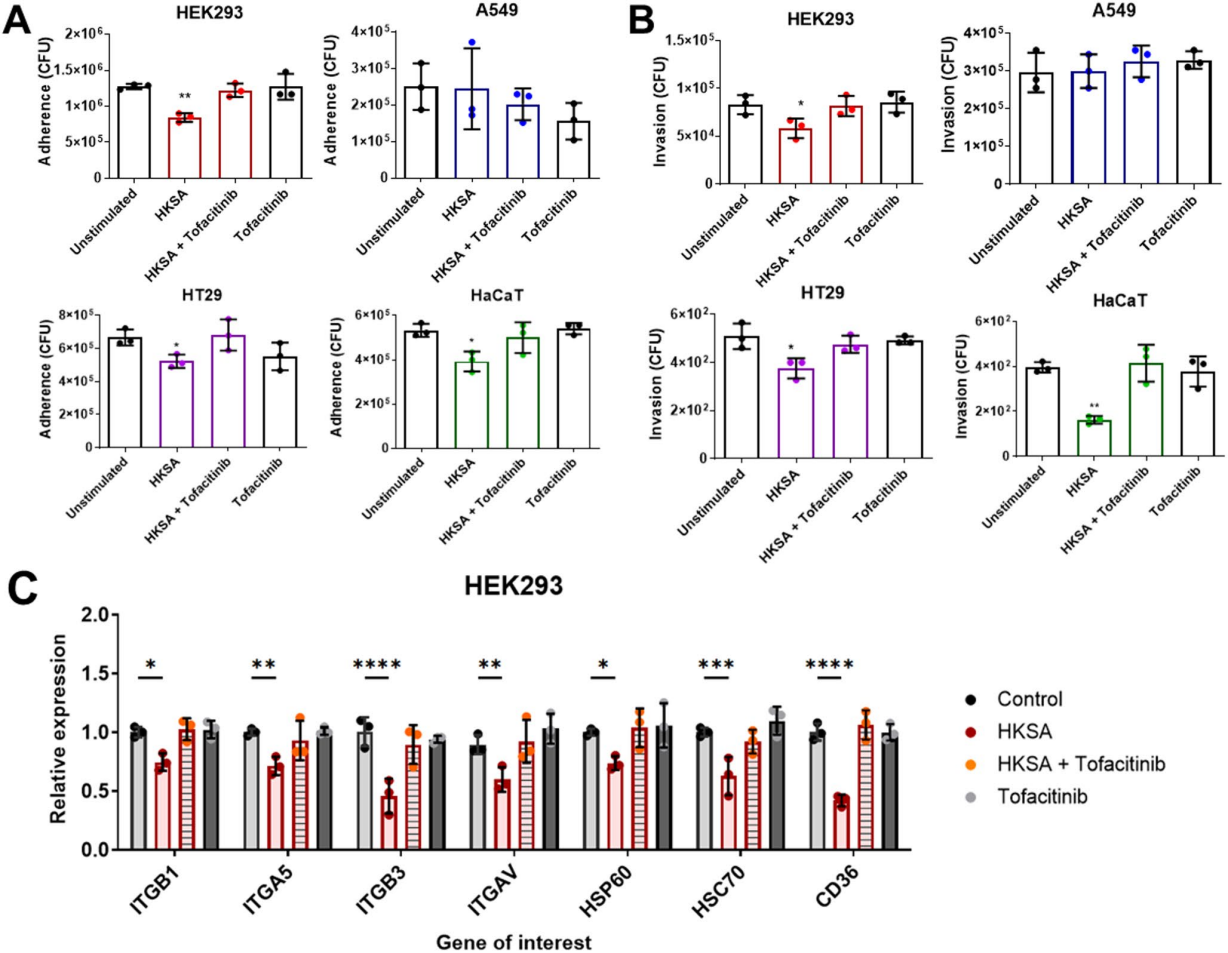
Tofacitinib abrogated the effect of the cytokine cocktail produced by Monomac-6 cells. (**A**) Adherence and (**B**) invasion assays were performed using *S. aureus* USA300 LAC and overnight pretreated cells with the supernatant of HKSA-stimulated Monomac-6 cells and unstimulated one and with the supplementation of tofacitinib (200 nM). (**C**) qPCR gene expression analysis of ITGB1, ITGA5, ITGB3, ITGAV, HSP60, HSC70 and CD36 of HEK293 cells was conducted using the same conditions as in adherence and invasion assays. For all the graphs, each data point represents the mean value ± SD (*n* = 3). Ordinary one way ANOVA were used to analyze the data from the adherence and invasion experiments followed by the Dunnett correction for multiple comparisons, and two-way ANOVA was used to analyze the data from the qPCR gene expression analysis, followed by the Bonferroni correction for multiple comparisons: ^*^p < 0.05; ^**^p < 0.01; ^***^p < 0.001; and ^****^p < 0.0001. Unstimulated: pretreated with supernatant of unstimulated Monomac-6 cells; HKSA: pretreated with supernatant of stimulated Monomac-6 cells with HKSA at MOI of 30; HKSA + Tofacitinib: pretreated with supernatant of stimulated Monomac-6 cells with HKSA at MOI of 30 and tofacitinib (200 nM); Tofacitinib: pretreated with supernatant of unstimulated Monomac-6 cells treated with tofacitinib (200 nM)

For the adherence and invasion assays, we supplemented the cell culture medium with 200 nM tofacitinib together with the supernatant of HKSA-stimulated Monomac-6 cells. In the adherence and invasion assays, we observed a similar effect as that of dexamethasone, which resulted in similar adherence and invasion rates of *S. aureus* as the unstimulated or control groups. Tofacinib supplementation abrogated the effects of the cytokine cocktail in the supernatant of HKSA-stimulated Monomac-6 cells on decreasing *S. aureus* adherence to and invasion into host cells, which was observed in HEK293, HT29 and HaCaT cells but not in A549 cells (Fig. 5A, 5).

We also measured the relative gene expression of ITGB1, ITGA5, ITGB3, ITGAV, HSP60, HSC70, and CD36 in the treated HEK293 cells with and without tofacitinib. We chose these genes because they were significantly decreased in HEK293 cells treated with the supernatant of HKSA-stimulated Monomac-6 cells (Fig. 4). The experiments revealed that supplementation with tofacitinib abrogated the impact of the cytokine cocktail on altering receptor expression levels in the cells. As a result, the receptor expression levels remained similar to those in the control group (Fig. 5C). These results confirmed the decrease in the expression of receptors for *S. aureus* adhesins and invasins in host cells, which was followed by the reduced adherence and invasion observed in HEK293, HT29 and HaCaT cells due to the activity of the cytokines released by Monomac-6 cells stimulated by HKSA.

## Discussion

Our results demonstrate that cytokines produced in response to *S. aureus* exposure play an important role in reducing bacterial adherence and invasion into nonphagocytic host cells. Monomac-6 cells stimulated with heat-killed *S. aureus* (HKSA) released a cytokine cocktail rich in pro-inflammatory cytokines, such as TNF-α, IL-1β, IL-6, and IL-8, and anti-inflammatory cytokine, IL-10, which significantly decreased the adherence and invasion of *S. aureus* in HEK293, HT29, and HaCaT cells. Pretreatment of the host cells using individual cytokines resulted similar tendency, in general, hinder bacterial adherence and invasion into host cells.

The finding that individual cytokines differentially affected *S. aureus* adherence and invasion across various cell types highlights the complexity of cytokine signaling in host-pathogen interactions [73]. In HEK293 cells, most of the cytokines tested reduced both bacterial adherence and invasion, whereas in HT29 cells, specific cytokines, such as TNF-α and IL-6, primarily affected bacterial adherence, while IFN-γ and IL-1β impacted both adherence and invasion. These observations might indicate that the efficacy of cytokines in modulating bacterial invasion may depend on the specific cell type and the expression of key receptors involved in bacterial adhesion and invasion.

Further investigation showed the pretreatment of host cells with cytokine cocktail lead to reduced expression of host cell receptors for *S. aureus* adhesins and invasins. As depicted in Fig. 4, the expression of integrin α5β1 (ITGA5 and ITGB1), the receptors for FnBPs-fibronectin, a primary mechanism of *S. aureus* invasion, were significantly reduced in HEK293, HT29, and HaCaT, but not in A549 cells. This reduction is consistent with the adherence and invasion results, suggesting that the cytokine cocktail inhibits *S. aureus* invasion by altering receptor expression. Genes encoding integrins (ITGB1, ITGA5), heat shock proteins (HSP60, HSC70), and annexin (ANXA2) were significantly downregulated in cells pretreated with the cytokine cocktail, which corresponded to the observed decrease in *S. aureus* adherence and invasion. The alteration of receptor expression is due to the activation of JAK-associated receptors by cytokines, confirmed by the supplementation of tofacitinib, a JAK inhibitor, reversed the effects of cytokine cocktails, which leads to JAK-STAT signaling cascade activation and the transcriptional response via the repression or activation of target genes [74]. These results suggest that cytokines hinder *S. aureus* immune evasion by altering the expression of host cell receptors, reducing the availability of the receptors for adhesins and invasins and limiting bacterial invasion and persistence inside host cells.

A decrease in adherence and invasion was observed in all cell types except for the lung epithelial cells, represented by the A549 cell line in this study. Consistent with this, the internalization rate of *S. aureus* in A549 cell line was observed to be 10- to 1000- fold higher compared to the other cell lines used in this study. This indicates that while other tested cell lines showed an active internalization of *S. aureus*, A549 cell line exhibited a substantially higher internalization. The high efficiency in bacterial internalization is may be due to the ability of airway epithelial cells to actively internalize bacteria as part of their host defense mechanism against inhaled pathogens although it could ultimately damage host cells due to incomplete killing and intracellular replication [52, 75, 76]. The receptor expression pattern also explain why we did not observe a significant difference in the group pretreated with the supernatant containing the cytokine cocktail compared with the control group. The high expression levels of ITGB3 and HSC70 might compensate the effect of the lower expression levels of HSP90, HSP60 and CD36 in A549 cells. The diverse effects of cytokines across the cell types in influencing the transcription level of target genes, as we observed in this study, were due to the pleiotropic and redundant functions among the cytokines, which are strongly dependent on the presence of other signaling components and cell type-specific receptor usage [77, 78].

While our study provides valuable insights into the role of cytokines in modulating *S. aureus* adherence and invasion using established cell lines, we acknowledge that the phenotypic responses observed in these immortalized cell lines may differ from primary human cells. Cell lines can exhibit altered signaling pathways, receptor expression profiles, and responsiveness to environment compared to the primary cell lines [79–81]. In addition, this study specifically focused on cytokine-mediated changes in host surface receptor expression involved in bacterial adhesion and entry. We did not examine the potential effects of cytokines on intracellular bacterial survival or replication following invasion. Future studies involving primary epithelial cells or organotypic culture systems would provide more physiologically relevant data and help validate the mechanisms identified in this model system.

In conclusion, our findings highlight the important role of cytokines in inhibiting *S. aureus* adherence and invasion into nonphagocytic cells. By modulating host cell receptor expression and limiting bacterial adhesion and invasion, cytokines provide a crucial line of defense against intracellular *S. aureus* survival and recurrence of infection.

## Electronic Supplementary Material

Below is the link to the electronic supplementary material.

Supplementary Material 1

## Data Availability

No datasets were generated or analysed during the current study.
