# Supplementary material for: Cytokine-mediated inhibition of Staphylococcus aureus adherence and invasion into nonphagocytic cells
Source: Med Microbiol Immunol. 2025 Jun 20;214(1):31. doi: 10.1007/s00430-025-00840-4 (PMC12181134; doi:10.1007/s00430-025-00840-4)
Supplement: Supplementary file 1 — Supplementary Material 1 [file 430_2025_840_MOESM1_ESM.docx]

**Supplementary Materials**

**Supplementary Table 1.** Primers used for the qPCR analysis of genes of interest.

| **Gene** | **Sequence (5’ – 3’)** | **Target Gene** |
| --- | --- | --- |
| ITGB1 F | CCGCGCGGAAAAGATGAATTTACAA | Integrin beta 1 |
| ITGB1 R | TCATCTGGAGGGCAACCCTTCT | Integrin beta 1 |
| ITGA5 F | CAGGGTCGGGGGCTTCAACT | Integrin alpha 5 |
| ITGA5 R | AGCCGAGAGCCTTTGCTGTCA | Integrin alpha 5 |
| ITGB3 F | CCCATGAGTTGGCTGGGAAT | Integrin beta 3 |
| ITGB3 R | TGCCCAAGTCTGTGTGGTTT | Integrin beta 3 |
| ITGAV F | CGCACTTCGGCGATGGCTTTT | Integrin alpha V |
| ITGAV R | GAAACATCCGGGAAGACGCGCTG | Integrin alpha V |
| HSP90A F | TCCTCGCCGCCGTTTCTGAG | Heat shock protein 90 |
| HSP90A R | CCTCAGGCATCAGTAGCCTAAGCA | Heat shock protein 90 |
| ANXA2 F | CGCACGGCCCAGGTTATCTTGTAG | Annexin |
| ANXA2 R | CAAAGCATCCCGCTCAGCATCA | Annexin |
| VWF F | GGGAGGGTGGTTGGTGGATGT | von Willebrand factor |
| VWF R | GCAGGAATCATCTTCCCCTGCAAAT | von Willebrand factor |
| DSG1 F | ACAGAGCCCGTTGTTAGTGG | Desmoglein |
| DSG1 R | TCTCGCAAGTCAGGCATCTC | Desmoglein |
| HSP60 F | CGCCGCCCCGCAGAAATG | Heat shock protein 60 |
| HSP60 R | ACTGTTCTTCCCTTTGGCCCCAT | Heat shock protein 60 |
| HSC70 F | CGCCTGCAGCTCTTGGGTTT | Heat shock 70 |
| HSC70 R | ACGTTTGGCATCAAAAACTGTGTTG | Heat shock 70 |
| CD36 F | CAGATGCAGCCTCATTTCCACCT | CD36 |
| CD36 R | ACACAGGTCTCCCTTCTTTGCATT | CD36 |
| B2M F | GCAGCATCATGGAGGTTTGAAGATG | beta-2-microglobulin |
| B2M R | ACCTCTAAGTTGCCAGCCCTCC | beta-2-microglobulin |


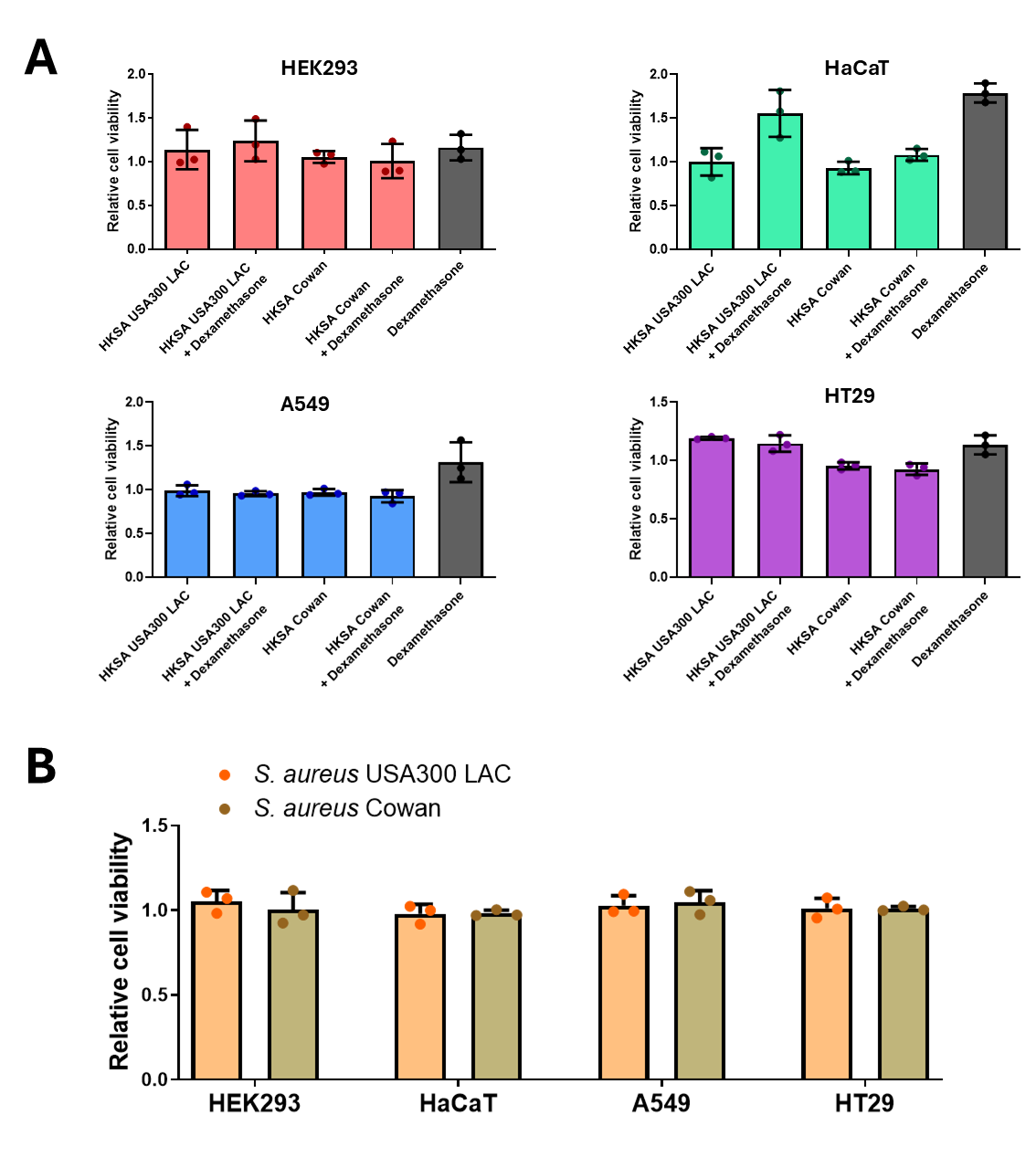


**Supplementary Figure 1. The cytokine cocktail in the Monomac-6-stimulated supernatant and living *S. aureus* cells did not decrease cell viability. (A)** The cells were pretreated with supernatant from Monomac-6 cells supplemented with HKSA, HKSA + dexamethasone (50 μg/ml), dexamethasone alone and untreated as a control. After overnight pretreatment, we measured the cell viability of each group and normalized it to that of the control group. **(B)** The cells were co-cultured with *S. aureus* USA300 LAC and Cowan at MOI of 30 and incubacted for 1.5 h. Gentamicin was added into the wells afterwards to kill *S. aureus* prior the cell viability measurement. We did not observe any significant reduction in cell viability in any of the 4 cell lines. For all the graphs, each data point represents the mean value ± SD (n = 3).


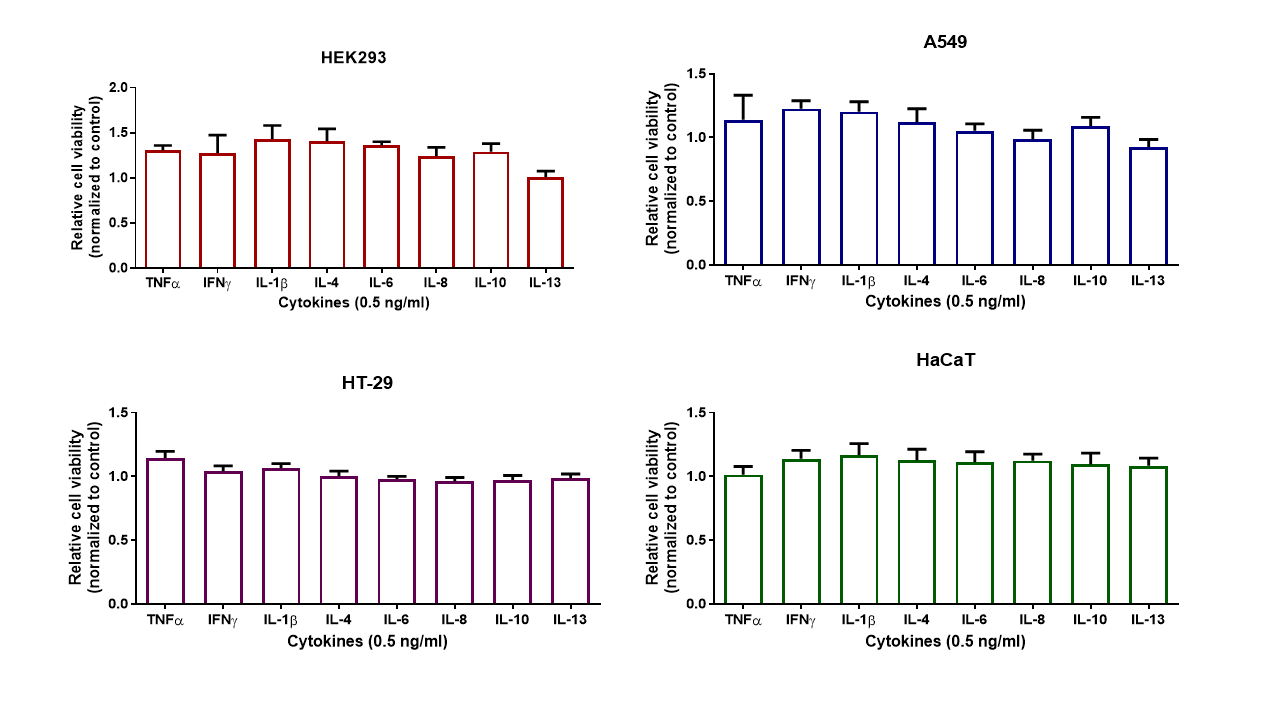


**Supplementary Figure 2. Individual cytokines did not affect cell viability.** The cells were pretreated overnight with individual cytokines (TNFα, IFNγ, IL-1β, IL-4, IL-6, IL-8, IL-10, and IL-13) at a concentration of 0.5 ng/ml prior to the assays and without any cytokines as a control. and untreated as a control. After overnight pretreatment, we measured the cell viability of each group and normalized it to that of the control group. We did not observe any significant reduction in cell viability in any of the 4 cell lines. For all the graphs, each data point represents the mean value ± SD (n = 3).
